# Immunity to α-Gal: The Opportunity for Malaria and Tuberculosis Control

**DOI:** 10.3389/fimmu.2017.01733

**Published:** 2017-12-04

**Authors:** Alejandro Cabezas-Cruz, José de la Fuente

**Affiliations:** ^1^UMR BIPAR, INRA, ANSES, Ecole Nationale Vétérinaire d’Alfort, Université Paris-Est, Paris, France; ^2^Faculty of Science, University of South Bohemia, České Budějovice, Czechia; ^3^Institute of Parasitology, Biology Center, Czech Academy of Sciences, České Budějovice, Czechia; ^4^SaBio, Instituto de Investigación en Recursos Cinegéticos IREC (CSIC-UCLM-JCCM), Ciudad Real, Spain; ^5^Department of Veterinary Pathobiology, Center for Veterinary Health Sciences, Oklahoma State University, Stillwater, OK, United States

**Keywords:** α-Gal, blood groups, infectious diseases, probiotic, vaccine

## Infectious Diseases, a Challenge to Modern Medicine

Among all infectious diseases, malaria and tuberculosis constitute leading causes of morbidity and mortality of human populations in developed and undeveloped countries (1, 2). In 2015, the WHO reported that 10.4 million people had tuberculosis and 1.8 million of them died from the disease (1). Despite a reduction of malaria cases between 2000 and 2015 (3), the WHO reported 212 million cases and 429,000 deaths due to this disease in 2015 alone (2). Drug resistance to first-line antimalarial drugs (e.g., chloroquine, sulfadoxine–pyrimethamine, and artemisinin) is a major constrain of malaria control Sub-Saharan Africa (4). Likewise, multidrug-resistant tuberculosis is a growing problem worldwide (5). Thus, the control of these diseases is among the most challenging tasks of public health worldwide. Drug overuse and misuse are recognized as the main drivers of drug resistance in parasites and pathogenic bacteria (4, 6). The identification of genetic factors affecting the susceptibility to these infectious diseases is essential toward reducing drug overuse and inappropriate treatment regimes. In this opinion, we propose that blood groups, a major driver of anti-α-Gal immunity and malaria and tuberculosis incidence (7), can be used to tailor anti-malaria and anti-tuberculosis vaccination. Blood group A and O individuals, that can potentially develop strong anti-α-Gal immunity (8), could be immunized with probiotic-based vaccines to enhance the natural levels of anti-α-Gal antibodies. This immunity could lead to protection against these diseases which in turn would reduce the use of anti-malaria and anti-tuberculosis drugs.

## Blood Groups, Infectious Diseases, and Anti-α-Gal Immunity

**Figure 1:**
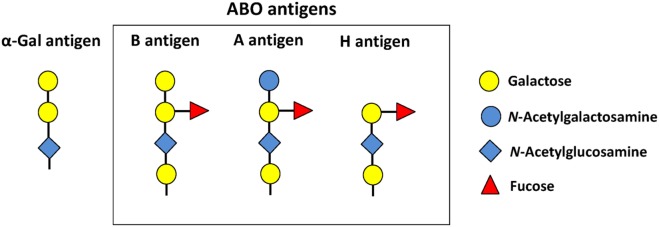
Glycan structure of blood group antigens and α-Gal. Blood type B, A, and O individuals express the B, A, and H antigens, respectively. Adapted from Ref. (8, 9).

The ABO histo-blood groups consist of two antigens (A and B), and four blood types (A, B, AB, and O) of which blood types A, B, and O are the most frequent among human populations, being the O type the most common (9). The blood type O results from the homozygous inheritance of two null ABO alleles and individuals in this group express the antigen H, the precursor of blood types A and B (Figure 1). The ABH antigens are carbohydrates attached to glycosphingolipids and glycoproteins. In general, humans have antibodies against missing A or B antigens (9). Therefore, individuals with blood type A have antibodies against antigen B, but not against self-antigen A (9). Individuals with blood type O have antibodies against both A and B antigens (9). The ABO blood type correlates with the susceptibility and severity of malaria and tuberculosis (9–11). However, so far, most of the mechanisms relating ABO blood types to infectious diseases are based on host cell–pathogen interactions (12, 13). For example, blood type O protects against severe malaria caused by *Plasmodium falciparum* through the mechanism of reduced rosetting (i.e., spontaneous binding of infected erythrocytes to uninfected erythrocytes) (12). By contrast, blood type A individuals are more susceptible to severe malaria because the *P. falciparum*–encoded repetitive interspersed families of polypeptides, expressed on the surface of infected red blood cells, binds more efficiently blood group A and increases the rosetting (13). Recent findings, however, showed that gut microbiota induces a protective immune response against malaria transmission by mosquitoes (14). This mechanism was associated to the antigen Galα1-3Galβ1-(3)4GlcNAc-R (α-Gal) expressed by microbiota bacteria and all mammals, but no by Old World monkeys, apes, and *Homo sapiens* (14, 15). In particular, humans have three frame-shift mutations in the gene (*GGTA1*) encoding for the enzyme α1,3-galactosyltransferase (α1,3GT) that result in premature stop codons truncating the α1,3GT enzyme which prevents the synthesizes of the carbohydrate α-Gal (15). Therefore, human cells lost the ability to produce this carbohydrate, which resulted in an almost unique capacity to produce high antibody titers against α-Gal (14, 15). Anti-α-Gal antibodies (IgM and IgG) induced by gut microbiota inhibited *Plasmodium* (i.e., *P. berghei* and *P. yoelii*) transmission by *Anopheles* mosquitoes, with a negative correlation between the levels of anti-α-Gal antibodies and the incidence of *P. falciparum* infection in human populations of endemic regions (7, 14). Individuals from Mali and Senegal exposed to mosquito bites were not infected by *P. falciparum* when having high anti-α-Gal antibody levels (7, 14). Strikingly, anti-α-Gal humoral response in *GGTA1* gene knockout (KO) mice provides “*sterilizing immunity”* against *Plasmodium* sporozoites transmission by mosquitoes (14). Sterilizing immunity “*is a unique immune status, which prevents effective pathogen infection into the host and is different from the immunity that allows infection but with subsequent successful eradication of the pathogen”* (16). Particularly, gut colonization by *Escherichia coli* O86:B7, which expresses α-Gal, blocked *Plasmodium* infection in 60% of the mice (14). This was not the case when *GGTA1* KO mice were or were not colonized by *E. coli* K12 that do not express α-Gal (14). Likewise, α-Gal immunization, with a TLR9 agonist adjuvant, enhanced the levels of anti-α-Gal antibodies and reduced the risk of *Plasmodium* infection by 88% compared to 61% risk reduction without adjuvant (14). Furthermore, α-Gal immunization arrested the transit of sporozoites from the skin into the liver, without interfering with sporozoite inoculation by mosquitoes. The cytotoxic effect of anti-α-Gal antibodies was restricted to the mice dermis and was dependent on the classical pathway of complement activation (14). It is important to note that the parasitemia, disease severity, and mortality were similar among those *GGTA1* KO mice that were infected regardless of gut colonization by *E. coli* O86:B7 or α-Gal immunization. This suggested that α-Gal immunity protects against *Plasmodium* transmission, but not against the erythrocytic stage of this parasite (14). Thus, anti-α-Gal immunity, if effective at the population level, has the potential to influence malaria incidence, but not disease severity or protection once the disease is established.

Likewise, tuberculosis patients in the Iberian Peninsula (Portugal and Spain) had low anti-α-Gal antibody levels when compared to healthy individuals (7). These groundbreaking findings suggested that anti-α-Gal antibodies might protect not only against *Plasmodium* parasites but also against other pathogens expressing α-Gal on their surface (17, 18). Remarkably, several pathogens such as *Plasmodium* spp. (14), *Mycobacterium marinum* (closely related to *Mycobacterium ulcerans* and *Mycobacterium tuberculosis*) (7), *Leishmania* spp. (19), and *Trypanosoma* spp. (20, 21) were reported to produce and express α-Gal on their surface, and thus anti-α-Gal antibodies could control their infection by complement-mediated lysis (14). The current paradigm is that immunity against *M. tuberculosis* relies exclusively on cellular defense mechanisms (22). However, mounting evidence supports that humoral immunity contributes to protection against tuberculosis (22, 23). In agreement with a protective role of antibodies against *M. tuberculosis*, Costello et al. (24) reported that antibody response to the glycolipid lipoarabinomannan limited bacteria dissemination in childhood tuberculosis. And passive immunotherapy using antibodies against different antigens has been shown to be protective in experimental models of tuberculosis (25).

Notably, the structure of blood type B [Galα1-3(Fucαl,2)Gal] is very similar to antigen α-Gal (Figure 1) because they share the disaccharide Galα1-3Gal (gal2) (8). In addition, gal2 is a crucial and sufficient epitope for anti-α-Gal antibody recognition (26). Accordingly, individuals with blood type B have a reduced antibody response against the related antigens α-Gal, gal2, and the blood antigen B (8). This lead us to the hypothesis that self-tolerance to blood type B affects the immune response to α-Gal, which in turn affects the susceptibility to infectious diseases caused by pathogens carrying α-Gal on their surface (7). The direct association between blood type B, low anti-α-Gal antibody titers, and the susceptibility to pathogens carrying α-Gal on their surface remains to be fully verified. However, this hypothesis was partially tested by correlation analysis between the incidence of malaria, tuberculosis, and dengue and the frequency of ABO blood types in endemic regions (7). The frequency of blood type B was positively correlated with the incidence of malaria and tuberculosis, but not with the incidence of dengue (7). By contrast, a negative correlation was observed between the frequency of blood type A and the incidence of malaria and tuberculosis (7). Both *Plasmodium* spp. and *Mycobacterium* spp. contain α-Gal on their surface, while Dengue virus does not produce this antigen (7, 14). In agreement with these results, a 4-year prospective cohort study in childhood malaria in Mali showed that children having blood types B and AB had higher incidence rate (blood type B: 1.63 and blood type AB: 1.65) compared to those children with blood types A and O (blood type A: 1.57 and blood type O: 1.45) (11). Other studies in endemic regions supported the association between blood type B and high incidence, prevalence, or severity of malaria (27, 28). Similar results were published for tuberculosis (10).

Bhatt et al. (3) reported that malaria control strategies have had a dramatic effect on malaria incidence in sub-Saharan Africa by reducing the incidence of clinical disease by 40% between 2000 and 2015. Interestingly, we found that the reduction in malaria incidence per country from 2000 to 2015 was negatively correlated with the frequency of blood type B (7). This finding suggests that the control of malaria has been less effective in countries with the highest frequency of blood type B, and therefore more susceptible individuals. Collectively, these results have important implications for the control of infectious microorganisms containing α-Gal on their surface.

## Microbiota, Infectious Diseases, and Anti-α-Gal Immunity

**Figure 2:**
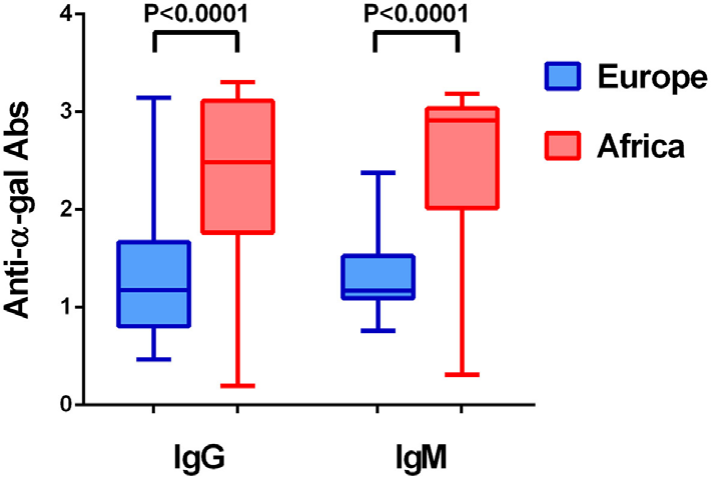
Level of anti-α-Gal IgM and IgG antibodies in healthy individuals from Africa and Europe. The figure displays the level of anti-α-Gal IgM and IgG in individuals from Senegal (Africa) and Portugal and Spain (Iberian Peninsula, Europe). Anti-α-Gal IgM and IgG antibody levels (O.D. 450 nm) were determined by ELISA in sera from healthy adults (7). The level of both immunoglobulins was significantly higher in African individuals.

In addition to blood group, gut microbiota composition has also been associated with malaria and tuberculosis. A recent study showed that cecal content transplants from malaria “resistant” or “susceptible” mice to germfree mice resulted in low and high *Plasmodium* spp. burdens, respectively (29). Further microbiota composition analysis revealed increased abundance of *Lactobacillus* and *Bifidobacterium* in resistant mice demonstrating that gut microbiota shaped the severity of malaria (29). In agreement with the protective role of *Bifidobacterium* against malaria severity, the gut microbiota of Malian children at lower risk of *P. falciparum* infection contained a significantly higher proportion of *Bifidobacterium, Streptococcus*, and Enterobacteriaceae (i.e., *Escherichia* and *Shigella*) compared to subjects at higher risk of *P. falciparum* infection (30). Gut microbiota composition is very different between malaria endemic and non-endemic countries (31). Contrasting microbiota composition can be due to differences in diet (31), but also to host–pathogen adaptations, in which individual from endemic countries acquired, maintain and develop a gut microbiota that may influence protection to malaria transmission and/or tolerance to severe malaria. Interestingly, production of anti-α-Gal antibodies in humans is thought to be driven by exposure to microbiota bacteria of the *Klebsiella* spp., *Serratia* spp., and *E. coli* spp. expressing α-Gal (32). As mentioned above, this has been experimentally tested and gut colonization by the human pathobiont *E. coli* O86:B7 elicited anti-α-Gal antibodies in *GGTA1* KO mice and in primates (33, 34). We hypothesized that contrasting microbiota composition between malaria endemic and non-endemic countries may have an effect in anti-α-Gal antibody levels. In fact, anti-α-Gal IgG and IgM antibody levels in healthy individuals from malaria endemic regions are significantly higher than those of individuals from non-endemic regions (Figure 2).

The experiments by Villarino et al. (29) were carried out in wild-type mice that express α-Gal and cannot develop anti-α-Gal antibodies. Therefore, the protective role of *Lactobacillus* and *Bifidobacterium* in these experiments was not related in any way to anti-α-Gal immunity. It is remarkable that Malian children microbiota is composed by both *Escherichia* spp. and *Bifidobacterium* (30). These two bacteria, when present in the same individual, may decreased simultaneously the risk of malaria infection and disease severity, respectively. Anti-α-Gal immunity triggered by *E. coli* gut colonization targets *Plasmodium* sporozoites in the skin immediately after mosquito transmission but once the parasites reach the blood, the anti-α-Gal antibodies are not effective (14). Thus, by reaching the blood, the parasites escape the anti-α-Gal immunity. In this scenario, the presence of *Bifidobacterium* in the gut microbiota can play an important role by decreasing malaria severity. Despite results by Villarino et al. (29) were not related to α-Gal immunity, the possibility that *Lactobacillus* and *Bifidobacterium* influence the response to α-Gal in humans cannot be rule out.

The role of gut microbiota in tuberculosis remains largely unexplored (35). However, a recent study found that antibiotic-induced dysbiosis increased significantly the bacterial burden in lungs and dissemination of *M. tuberculosis* to spleen and liver (36). Furthermore, microbiota diversity reconstitution by fecal transplantation significantly reduced the bacterial load in the lungs (36). Further studies should test whether *Bifidobacterium* and *Lactobacillus* play a protective role in tuberculosis as in malaria.

## Challenges of Developing a Probiotic-Based Vaccine

The carbohydrate α-Gal is a protective antigen (14, 19, 37, 38). α-Gal immunization induces an protective immune response against *Plasmodium* spp. (14)., *Trypanosoma cruzi* (37), and *Leishmania* spp. (19, 38). This provides the basis to develop a single-antigen pan-vaccine to control major infectious diseases (18). This prospective vaccine can be developed using classical approaches of antigen formulation and immunization by injection. For example, conjugation of α-Gal to carrier proteins such as bovine serum albumin (BSA) (14), α-Gal-containing neoglycoproteins covalently attached to BSA (38), and virus-like particles displaying the α-Gal carbohydrate (19) among others. However, the most innovative implication of the study by Yilmaz et al. (14) is the potential use of probiotic bacteria to elicit protective anti-α-Gal antibodies (39). Such approach would have obvious advantages considering that probiotic-based products are safe, easy to distribute, well received by the public, and with a well-established regulatory body (40). Currently, research on the use of probiotics to induce anti-α-Gal immunity is very limited. Initial reports showed that consumption of fermented milk containing *Lactobacillus casei*, which express α-Gal, in healthy adults did not change anti-α-Gal antibody levels (41). However, this preliminary report does not allow concluding that developing an α-Gal probiotic-based vaccine is an impossible task. The diversity of best known probiotics *Lactobacillus* (42), *Lactococcus* (43), and *Bifidobacterium* is astonishing (44). For example, the genus *Lactobacillus* has 154 validly described species and 19 subspecies (42). Exploring such diversity may render promising bacterial species candidates that express α-Gal and may also enhance the immunity to this antigen. The *GGTA1* KO mice would be a relevant model in these studies. A difficulty may arise when developing a probiotic-based vaccine using Gram-positive bacteria. The α-Gal in members of the family Enterobacteriaceae (Gram-negative bacteria, e.g., *E. coli* spp.) is mainly associated with the bacterial capsule and cell wall glycoproteins, as well as with carbohydrate units of bacterial lipopolysaccharide (LPS) (32). The association of α-Gal to highly immunogenic components such as LPS, not present in Gram-positive bacteria, may influence the immune response elicited against this carbohydrate in the intestinal mucosa. Toll-like receptor (TLR) 4 for which LPS is a specific, and powerful, activator may play a role in the immunity against α-Gal associated with LPS. Thus, as previously proposed (39), probiotic-based vaccines using Gram-positive bacteria may be combined with TLR4 agonist. A way to implement this is to transform the candidate Gram-positive bacteria with a plasmid containing LPS specific peptide mimotopes (45). This LPS mimotopes are short peptide sequences of seven amino acids that activate TRL4 signaling pathway and trigger the secretion of inflammatory cytokines by macrophages (45). Gram-positive bacteria co-expressing LPS mimotopes and α-Gal have the potential to overcome the low antigenicity of α-Gal expressed by *L. casei* (41). Alternatively, probiotic Gram-negative bacteria such as *E. coli* Nissle 1917 strain (46) can be used. Whether *E. coli* Nissle 1917 expresses α-Gal or not is currently unknown. If these bacteria do not express α-Gal naturally, they can be transformed with a plasmid containing bacterial α-1,3-galactosyltransferase reported in *E. coli* (47) and other bacteria (48).

## Concluding Remarks

The identification of anti-α-Gal immunity as an important factor in malaria transmission (14), together with the finding that blood type B decreases anti-α-Gal antibody levels increasing the susceptibility to malaria and tuberculosis (7, 8), can be used to implement specific measures for disease control. First, blood type B may be considered as a risk factor to develop malaria and tuberculosis. Second, probiotic-based vaccines can be used to induce a protective anti-α-Gal immunity in blood type A and O individuals. This vaccine has the potential to induce a long-lasting protective response against various highly prevalent infectious diseases such as malaria and tuberculosis caused by pathogens with α-Gal on their surface. Probiotic-based vaccines could rely on α-Gal-producing bacteria such as *Lactobacillus* spp. transformed with LPS mimotopes to activate TLR4. Alternatively, this vaccine can be based on the probiotic Gram-negative bacteria *E. coli* Nissle 1917. This probiotic-based vaccine would have low production costs and would be easy to administer to high-risk populations in the poorest regions of the world.

## Author Contributions

All authors contributed equally to the realization of the present work.

## Conflict of Interest Statement

The research was conducted in the absence of any commercial or financial relationships that could be construed as a potential conflict of interest.
